# ACE2 and TMPRSS2 in human kidney tissue and urine extracellular vesicles with age, sex, and COVID-19

**DOI:** 10.1007/s00424-024-03022-y

**Published:** 2024-10-09

**Authors:** Marie Lykke Bach, Sara Laftih, Jesper K. Andresen, Rune M. Pedersen, Thomas Emil Andersen, Lone W. Madsen, Kirsten Madsen, Gitte R. Hinrichs, Rikke Zachar, Per Svenningsen, Lars Lund, Isik S. Johansen, Lennart Friis Hansen, Yaseelan Palarasah, Boye L. Jensen

**Affiliations:** 1 Unit of Cardiovascular and Renal Research, Department of Molecular Medicine, University of Southern Denmark, Odense, Denmark; 2 Department of Nephrology, Odense University Hospital, Odense, Denmark; 3 Department of Clinical Microbiology, Odense University Hospital, and Research Unit for Clinical Microbiology, University of Southern Denmark, Odense, Denmark; 4 Department of Infectious Diseases, Odense University Hospital, and Research Unit for Infectious Diseases, University of Southern Denmark, Odense, Denmark; 5 Unit for Infectious Diseases, Department of Medicine, Sygehus Lillebælt, Kolding, Denmark; 6 Department of Urology, Odense University Hospital, Odense, Denmark; 7 Department of Pathology, Odense University Hospital, Odense, Denmark; 8 Department of Clinical Microbiology, Rigshospitalet, Copenhagen, Denmark; 9 Unit of Inflammation and Cancer Research, Department of Molecular Medicine, University of Southern Denmark, Odense, Denmark

**Keywords:** SARS-CoV-2, Angiotensin II, Exosomes, Hypertension, Albuminuria, Protease

## Abstract

SARS-CoV-2 virus infects cells by engaging with ACE2 requiring protease TMPRSS2. ACE2 is highly expressed in kidneys. Predictors for severe disease are high age and male sex. We hypothesized that ACE2 and TMPRSS2 proteins are more abundant (1) in males and with increasing age in kidney and (2) in urine and extracellular vesicles (EVs) from male patients with COVID-19 and (3) SARS-CoV-2 is present in urine and EVs during infection. Kidney cortex samples from patients subjected to cancer nephrectomy (male/female; < 50 years/˃75 years, *n* = 24; ˃80 years, *n* = 15) were analyzed for ACE2 and TMPRSS2 protein levels. Urine from patients hospitalized with SARS-CoV-2 infection was analyzed for ACE2 and TMPRSS2. uEVs were used for immunoblotting and SARS-CoV-2 mRNA and antigen detection. Tissue ACE2 and TMPRSS2 protein levels did not change with age. ACE2 was not more abundant in male kidneys in any age group. ACE2 protein was associated with proximal tubule apical membranes in cortex. TMPRSS2 was observed predominantly in the medulla. ACE2 was elevated significantly in uEVs and urine from patients with COVID-19 with no sex difference compared with urine from controls w/wo albuminuria. TMPRSS2 was elevated in uEVs from males compared to female. ACE2 and TMPRSS2 did not co-localize in uEVs/apical membranes. SARS-CoV-2 nucleoprotein and mRNA were not detected in urine. Higher kidney ACE2 protein abundance is unlikely to explain higher susceptibility to SARS-CoV-2 infection in males. Kidney tubular cells appear not highly susceptible to SARS-CoV-2 infection. Loss of ACE2 into urine in COVID could impact susceptibility and angiotensin metabolism.

**Supplementary information:**

The online version contains supplementary material available at 10.1007/s00424-024-03022-y.

## Introduction

The WHO estimates > 700,000,000 confirmed cases of coronavirus disease-19 (COVID-19) globally [83]. Along with comorbidities such as hypertension, chronic kidney disease (CKD) [8], diabetes mellitus (DM) [28, 61], high age, and male sex as well as high viral load have been identified as independent risk factors of severe disease [20, 52, 57]. While males and females have an equal prevalence of COVID-19, the higher prevalence of underlying comorbidities in males is believed to be one reason for the sex-related discrepancies in disease progression and severity [63]. Acute kidney injury (AKI) is common with an incidence of 20–40% in patients admitted to the intensive care unit (ICU) with COVID-19 [14, 34, 88]. The causal relation between age, male sex, severe acute respiratory syndrome coronavirus 2 (SARS-CoV-2), renal tropism, and AKI is unclear.

The SARS-CoV-2 virus uses angiotensin-converting enzyme 2 (ACE2) for cellular entry [46]. ACE2 is a transmembrane glycoprotein with an apparent molecular mass of 120 kDa and with a short C-terminal cytoplasmic tail and an extensively N-glycosylated N-terminal ectodomain containing the active site. ACE2 is more abundant in the kidney than in the lung, where it associates with the apical brush border of the proximal tubular epithelium [32, 66, 82]. ACE2 has activity in both membrane-bound and shed forms. Membrane-bound ACE2 degrades several angiotensin peptides with the highest affinity to the vasoconstrictor angiotensin II (ANGII), which is hydrolyzed to vasodilatory peptide angiotensin 1–7 [18, 64]. Membrane-bound ACE2 protein can be shed from cells by ADAM17 activity [43], and such ectodomain shedding releases a catalytically active and soluble form of ACE2 [42, 43]. In mice, soluble ACE2 is filtered at the glomerulus along with albumin [53]. It is unknown, whether ACE2 is lost into urine by enzymatic shedding or if ACE2 stays membrane-attached in released extracellular vesicles (EVs) from proximal tubular cells.

In addition to ACE2, the spike glycoprotein (S-protein) of SARS-CoV-2 requires the presence of host endogenous protease to cleave the S-protein before infection [56, 80]. The transmembrane protease serine 2 (TMPRSS2) co-localizes with ACE2 in lung epithelium and activates the SARS-CoV-2 S-protein, which enables virus-cell membrane fusion at the cell surface [35, 38, 67]. It is unclear if TMPRSS2 co-localizes with ACE2 in human kidney proximal tubules or if separate localizations explain observations of low primary infection in kidney epithelium [13]. SARS-CoV-2 RNA was detected postmortem in the renal tissue of patients with SARS-CoV-2 who died of acute respiratory distress syndrome with or without AKI [7, 17, 21, 58, 70]. On the other hand, kidney biopsies from patients with COVID-19 showed very low or no SARS-CoV-2 RNA and protein [2, 31, 41, 44, 65]. SARS-CoV-2 nucleoprotein (N-protein) has been reported in the urine of hospitalized patients and is associated with or without AKI [73, 78]. The isolation of urine extracellular vesicles (uEVs) could be used as a non-invasive “liquid” biopsy as uEVs are derived predominantly from the apical membrane and provide information on the integral membrane proteins and cytosolar proteins from the renal tissue. However, the use of EVs in patients with COVID-19 for virus recovery, for membrane protein investigation and as markers of kidney injury, is yet to be established [19, 69, 89].

The present studies were designed to address the following hypotheses: (1) In human kidney tissue, high age and male sex are associated with higher protein levels of ACE2 and TMPRSS2; (2) ACE2 and TMPRSS2 proteins co-localize in human kidney; (3) higher abundance of ACE2 and TMPRSS2 proteins in kidney tissue from males is reflected in higher abundance in urine EVs from patients with SARS-CoV-2 infection, and (4) SARS-CoV-2 antigen is present in urine and uEVs from patients admitted to the hospital with SARS-CoV-2 infection.

## Materials and methods

### Study design and population

#### Substudy 1: In human kidney tissue ACE2 and TMPRSS2 protein abundance associate with age and sex

Human renal tissue was obtained from patients undergoing nephrectomy due to renal cancer at the Department of Urology, Odense University Hospital, Denmark. All patients received oral and written information and gave written consent to participate. The use of tissue after consent was approved by the Ethics Committee of the Region of Southern Denmark (Project-ID: S-20140159) and the Danish Data Protection Agency (ID: 2012-58-0018). The tissue was selected to be as far away from the tumor as possible. Tissue samples were selected from the biobank within the 4 categories: male > 75 years, male < 50 years, female > 75 years, and female < 50 years (Supplemental Table 1). A second group of kidney cortex samples from male (*n* = 8) and female (*n* = 7) > 80 years was included.

#### Substudy 2: ACE2 and TMPRSS2 in uEVs from patients with SARS-CoV-2 infection and healthy control

Urine samples from patients (all > 60 years) with SARS-CoV-2 infection admitted to the hospital were collected at the Department of Infectious Disease at Odense University Hospital, Odense, Denmark (October 2020 through April 2021) (Project-ID: S-20200047C), approved by the Ethics Committee of the Region of Southern Denmark and the Danish Data Protection Agency (ID: 20/16202). Characteristics of included patients with COVID-19 are provided in Supplemental Tables 2–3. Disease severity was stratified based on the COVID-19 Treatment Guidelines provided by the National Institute of Health (Clinical Spectrum | COVID-19 Treatment Guidelines (nih.gov)). The inclusion of patients was prior the availability of COVID-19 vaccines. Based on the date of admission, the variant of SARS-CoV-2 can be estimated to be alpha according to Danish COVID-19 Genome Consortium (Danish COVID-19 Genome Consortium (covid19genomics.dk)). Urine samples were added protease inhibitor (cOmplete tab, Roche), one per 50 mL, directly after voiding and stored at − 80 °C. Spot urine samples from young healthy male controls were also added protease inhibitor. Samples were obtained from a previous study biobank (median age: 22.8 years) (Project ID: S-20150208) [87]. To study the significance of albuminuria without concurrent COVID, existing urine samples from kidney transplant recipients (KTRs) who displayed variable excretion of albumin in the urine were included (Project-ID: S-20150015) [33]. The study was carried out in accordance with the Declaration of Helsinki.

### Enzyme-linked immunosorbent assay (ELISA) for ACE2

ACE2 (human) sandwich ELISA kit (Cat. No. AG-45B-0023-KI01; AdipoGen Lifesciences) was used to determine ACE2 protein concentration in urine samples from COVID19 patients and in kidney cortex tissue homogenate samples from male and female > 80 years (*n* = 8 and 7). The plate was pre-coated with polyclonal antibody specific for human ACE2. The urine samples were loaded according to manufacturer’s instructions. The plate was developed with streptavidin-conjugated horseradish peroxidase (Strep-HRP) (1:2000), and 3,3′,5,′-tetramethylbenzidine (TMB), and stopped with 0.2 M H_2_SO_4_. The plate was read at 450 nm on a *V*_max_ microplate reader (VersaMax Plate Reader). A standard curve was generated by a curve fitting equation generated by standard curve fitting software (SoftMaxPro).

### Isolation of urine extracellular vesicles (uEVs)

Urine EVs were isolated by polyethylene glycol (PEG) precipitation as previously described [62]. 6 mL of urine from SARS-CoV-2 patients was thawed at 4 °C and centrifuged for 15 min at 5000 g (5910 R, Eppendorf) to remove cells and cellular debris. The supernatant was moved to a new tube with the addition of 5 mL freshly made 16% PEG6000 (Sigma-Aldrich) with 1 M NaCl and 1 cOmplete (Roche) tablet. Samples were incubated overnight (ON) at 4 °C and centrifuged for 15 min at 5000 g at 4 °C. The supernatant was removed, and the tubes were allowed to dry upside down for 10 min. The pellet was resuspended in 100 µL 1X phosphate-buffered saline (PBS) and mixed thoroughly. This procedure also precipitates SARS-CoV-2 virions [16].

### Immunoaffinity isolation

100 µL of isolated PEG precipitate was incubated with biotinylated ACE2 antibody (5 µg/mL) and in PBS (total 500 µL) in a rotator for 1 h at 4 °C. Samples were then incubated with streptavidin magnetic beads (Pierce, Thermo Fisher Scientific) for 60 min at 4 °C. The unbound fraction was removed and saved, and the beads were washed 3 times in 1000 µL of 1X PBS. The samples were eluted with 0.5% citric acid for 30 min and normalized with Tris (pH 7.4).

### Immunoblotting

Human renal cortex samples were homogenized in lysis buffer containing 20 mM Tris-HCL, 150 mM NaCl, 20 mM NaF, 10 mM Na_4_P_2_O_7_, 1% Triton X-100 (Merck), and protease inhibitor (cOmplete, Roche). Protein concentration was determined using Thermo Scientific™ Pierce™ Protein Assay (cat. No. 23209) using a serial dilution of bovine serum albumin as standard. Protein concentrations were used to normalize human renal cortex homogenates. Urine EVs were normalized to urine creatinine, where a concentration of 4 mmol/L of creatinine was considered significant. The samples were mixed with NuPAGE LDS Sample buffer (1/4) (Invitrogen) and NuPAGE Sample Reducing Agent (1/10) (Invitrogen) and separated on 4–12% Bis-Tris NuPAGE Gels in MOPS buffer (Invitrogen). The gel was blotted onto a polyvinylidene difluoride membrane (Immobilon-P, Merck Millipore) and subsequently blocked in TBST (20 mM Tris–HCl pH 7.6, 150 mM NaCl, 0.05% Tween-20) with 5% skim milk for 1 h at RT. Membranes were incubated at 4 °C overnight with primary rabbit anti-TMPRSS2 (1:2000, Cat. No. HPA035787, Sigma), rabbit anti-ACE2 (1:2000, Cat. No. SN0754, Novus Biologicals®), mouse anti-AQP1 (1:1000, B-11, Cat. No. sc-25287, Santa Cruz Biotechnology), mouse anti-ALIX (1:1000, 3A9, Cat. No. sc-53538, Santa Cruz Biotechnology), mouse anti-Na^+^-glucose transporter 2 (SGLT-2) (1:1000, D-6, Cat. No. sc393350, Santa Cruz Biotechnology), and mouse anti-CD63 (1:1000, Cat. No. sc-5275, Santa Cruz Biotechnology). The membranes were subsequently incubated with horseradish peroxidase-conjugated goat-anti-rabbit antibody (1:2000, Cat. No. P0448, Dako, Denmark) or goat anti-mouse antibody (1:2000, Cat. No. P0447, Dako, Denmark), 1 h at RT. The membranes were developed with enhanced chemiluminescence (PerkinElmer, Inc.) and recorded using Molecular Imager ChemiDOC™ XRS^+^ (Bio-Rad). For densitometry, band density was analyzed using Image Lab software (Bio-Rad). Homogenized kidney tissue (Regional Ethics Committee approval (project ID: S-20140159) was used as positive control. A pool of human renal cortex protein homogenate was deglycosylated using peptide: N-Glycosidase F (PNGase F) (Bio-Rad, cat. No. 170–6883), according to the manufacturer’s instruction. The denaturing substrate was added to the cortex homogenate and heated at 100 °C for 10 min. Samples were cooled to RT, and NP-40 was added before incubation with PNGase F at RT for 2 h. The samples were analyzed by immunoblotting as described above.

### Immunohistochemistry

Immunohistochemical labeling of tissue was performed on paraffin-embedded sections from patient kidneys removed due to cancer at the Department of Urology, Odense University Hospital, approved by the Regional Ethics Committee (project ID: S-20140159) and the Danish Data Protection Agency (ID: 2012-58-0018). Tissue sections were deparaffinized with Tissue Clear (Sakura ProHosp) and rehydrated in graded ethanol series (99.9–70%). Antigen retrieval was performed by boiling in TEG buffer (10 mM Tris, 0.5 mM EGTA, and pH 9.0) for 20 min. The sections were blocked in buffer (1xPBS 0.3%Triton X-100) and incubated with primary antibodies TMPRSS2 (1:100, HPA035787, Novus Biologicals®), ACE2 (1:1000, SN0754, Novus Biologicals®) diluted in blocking buffer. Negative control with the omission of primary antibodies was run in parallel. Staining was visualized with a solution of 3,3′-diaminobenzidine (DAB) (Cat. No. K3468, Dako), 1xPBS, and 30% H_2_O_2_ and counterstained with hematoxylin (Sigma-Aldrich). An Olympus BX51 microscope (Olympus, Tokyo, Japan) with a DP26 camera using cellSens software (Olympus) was used to take pictures.

### Detection of SARS-CoV-2

Every SARS-CoV-2 virion contains 1 SARS-CoV-2 RNA and about 1000 nucleoproteins [4], and after entering the host cell, a complementary negative sense genome length RNA is generated for amplification of plus strand virus genome RNA, as well as transcription of a series of plus strand subgenomic RNAs (sgRNAs) all with a common leader joined to gene sequences in the 3′-end of the virus genome [68]. Since the copy number of nucleoprotein is much higher than the SARS-CoV-2 RNA, both were tested by testing for both SARS-CoV-2 RNAs, sgRNAs, and the SARS-CoV-2 nucleoprotein.

### SARS-CoV-2 RT-qPCR

We tested for SARS-CoV-2 RNAs (nucleocapsid (N) gene and Envelope (E) gene), and the L452R mutation (found in the delta and omicron variants) and for the SARS-CoV-2 sgRNAs (Sub-Orf7A and Sub-E) using IVD certified assays (Pentabase, Odense, Denmark). The RT-qPCR was performed as previously described [37] with the modification that 200 µL urine was used instead of liquid Amies transport medium. Positive and negative controls were included in each run, and the supplied controls were diluted 1 + 3 in urine from non-infected patients to better mimic urine. This increased the Ct values by ~ 2.

### SARS-CoV-2 N-protein test

The N-protein in urine was measured using a quantitative Lateral Flow Immunoassay (LFIA) test (BioUhan, Solsten, Århus, Denmark). Detection limit 8.9 ng/L, CV% 8% at 45 ng/L. The assay is certified to measure N-protein in plasma and liquid amies swab medium. Therefore, N-protein dissolved in 5% Albumin /PBS (967 ng/L) was first diluted 5 times in urine before serial dilutions were made and measured in triplicate. Briefly, 12 µL N-protein urine was added to the test cassette, then 2 drops of reagent buffer were added; the strip was incubated for 15 min and finally read in the UV/IR reader. Sample loading was done using standard 20-µL laboratory pipettes. Each cassette contained a positive control strip.

### Data analysis and statistics

All data were tested for normal distribution using Anderson-Darlin, Shapiro-Wilks, and Kolmogorov–Smirnov’s tests. Non-normally distributed data were log-transformed. Two-way analysis of variance (ANOVA) followed by post-hoc Bonferroni or unpaired Student’s *t*-test was used as appropriate. To test correlation, linear regression with 95% confidence intervals was applied. A *p*-value < 0.05 was considered significant. Data are displayed as mean ± standard error of the mean (SEM), and log-transformed data are displayed as mean ± interquartile range. Analyses were carried out using GraphPad Prism 9.5.0.

## Results

### Immunoblotting and immunolabeling for ACE2 and TMPRSS2 with human kidney tissue

**Fig. 1:**
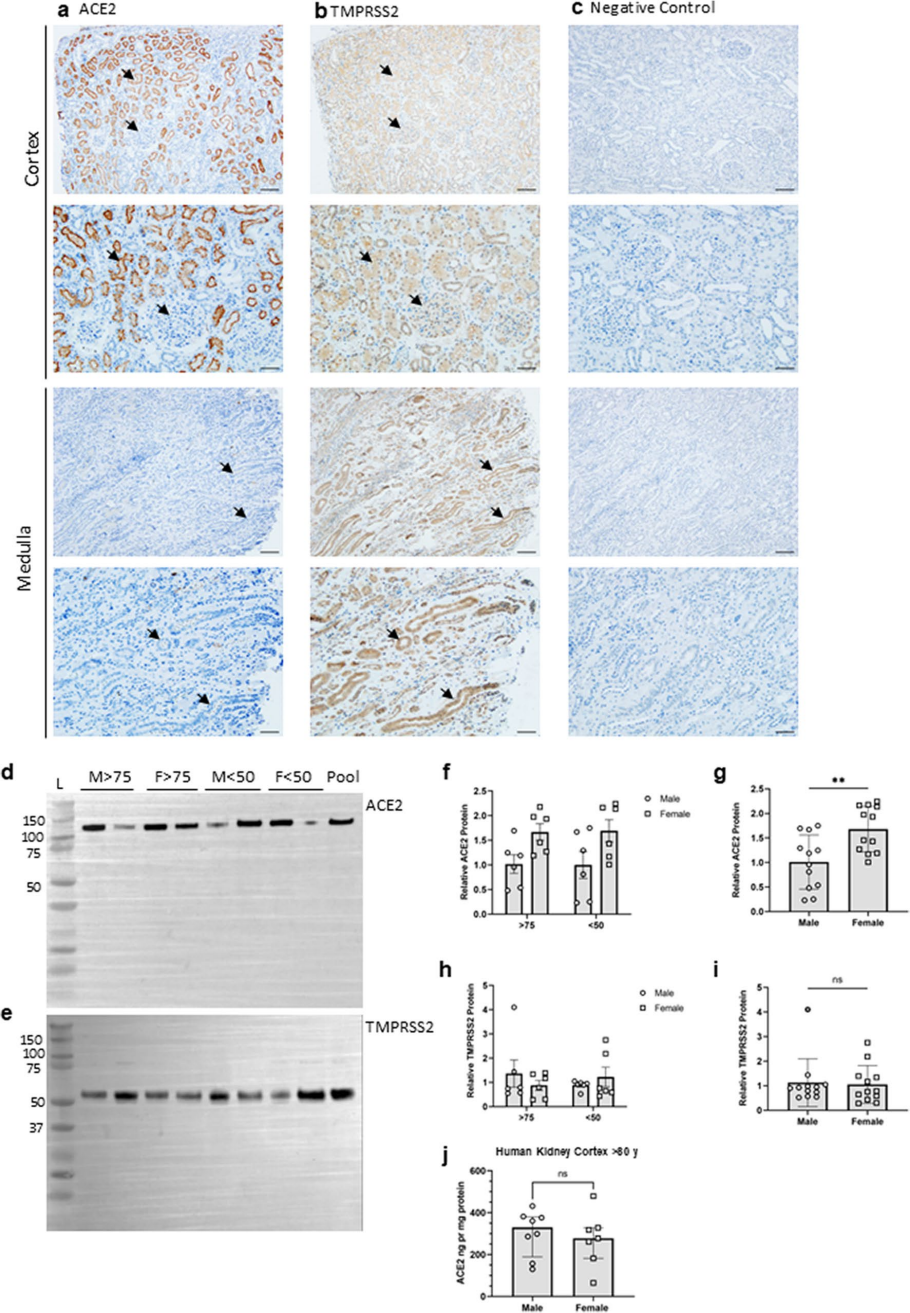
Detection of ACE2 and TMPRSS2 protein in human kidney tissue in relation to sex and age. Immunohistochemical labeling of adjacent human kidney sections for **a** ACE2 and **b** TMPRSS2 in cortex and medulla. Arrows indicate the same structure in adjacent sections from the same kidney. **c** Negative controls with omission of primary antibodies. ACE2 was observed only in kidney cortex, while TMPRSS was observed also in medulla. While ACE2 was strictly found in apical proximal tubular membranes, TMPRSS2 was seen in the cytoplasm with little overlap with ACE2. Immunoblotting results with kidney tissue homogenates for **d** ACE2 and **e** TMPRSS2; Lanes 1–2: Male (M) > 75 yr, lanes 3–4: female (F) > 75 yr, lanes 5–6: male < 50 yr, lanes 7–8: female < 50, yr and lane 9: positive control pool of human cortex. A single band appeared when probing with anti-ACE2 that migrated at ~ 120 kDa in lanes 1–9 while polyclonal, anti-TMPRSS2 primary antibody yielded band at ~ 54 kDa in lanes 1–9. **f** ACE2 and **h** TMPRSS2 protein abundances by densitometry normalized to the average of < 50yr male signal. There was no significant difference in the protein abundances of ACE2 and TMPRSS2 between age and sex. When data were pooled across age, **g** ACE2 protein abundance was significantly higher in tissue from females compared to males (*p* = 0.0049), while in **i** TMPRSS2 protein abundance was similar between males and females (*p* = ns). In **j**, ACE2 concentration (ng per mg protein) in tissue homogenate by ELISA from persons > 80yr showed no difference. Each bar represents the mean ± SEM. *p* < 0.05 was considered statistically significantly different, indicated with an asterisk (*). *n* = 6 for male > 75 yr, *n* = 6 for female > 75 yr, *n* = 6 for male < 50 yr, and *n* = 6 for female < 50 yr. L, ladder. scale bar: 100 µm

Immunoblotting of human kidney cortex homogenate revealed a protein migrating at ~ 120 kDa, exceeding the predicted molecular size of human ACE2 at 92 kDa (Supp.Fig. 1.a). Signal intensity correlated with protein load between 5 and 40 µg. Under non-reduced conditions, protein mobility remained unchanged, while deglycosylation shifted it to the predicted ~ 90 kDa (Supp.Fig. 1.b).

Immunoblotting for TMPRSS2 showed a single protein that migrated at the expected molecular size of the zymogen at 54 kDa in kidney cortex (5–40 µg protein) (Supp.Fig. 1.d). To further validate the TMPRSS2 antibody, cell lysate from transfected HEK293T cells overexpressing TMPRSS2 (#NBL1-17121), alongside an empty vector-transfected control cell lysate (Mock and NC), were subjected to SDS-PAGE-separation and western blot analysis (Supp.Fig. 1.e). The analysis revealed proteins migrating predominantly at 54kDa and weaker at 37kDa and 18 kDa under both reduced and non-reduced conditions. The mock HEK293T cell lysate could account for the additional, likely non-specific, bands observed in the overexpressing TMPRSS2 cell lysate (but never in tissue homogenate). In summary, the human kidney expresses glycosylated ACE2 protein and predominantly intact, likely zymogen, form of TMPRSS2.

Adjacent sections of human kidneys were labeled by immunohistochemistry for ACE2 and TMPRSS2 to evaluate the potential co-localization. ACE2 immunoreactive signal was associated predominantly with the apical side of the proximal convoluted tubules in the kidney cortex, characterized by the convolutions, basal nuclei and brush border, with no signal in the medulla (Fig. 1a). Weak but quite widely distributed immunoreactive signal for TMPRSS2 was observed in the renal cortex. The signal was more intense in the tubular epithelium in the medulla, likely collecting ducts (Fig. 1b). Comparison of adjacent sections showed co-localization in a minority of segments, likely proximal tubules in cortex. The labeling did not indicate co-localization of ACE2 and TMPRSS2 in the medulla. Negative controls without primary antibodies showed no signals (Fig. 1c). In summary, ACE2 and TMPRSS2 proteins are abundant in the human kidney, with no co-localization of immunolabeling signal in medulla and weakly in cortex.

### ACE2 and TMPRSS2 protein abundance in human kidneys with age and sex

To investigate if the protein levels of ACE2 and TMPRSS2 were higher in males and with increasing age, the protein abundance in renal cortex specimens was compared from patients below 50 years of age and above 75 years from both sexes (Fig. 1d, e). Patient age and distribution are provided in Supplemental Table 1. Aliquots from the same kidney cortex tissue homogenate pool served as standard controls in all blots. Semi-quantification by densitometric analysis showed no difference in protein abundance between groups for ACE2 and TMPRSS2 when normalized to the < 50 yr male group protein signal (Fig. 1f, h). When data were pooled across age, ACE2 protein abundance was slightly but significantly higher in females compared to males (*p* = 0.004) (Fig. 1g), while no significant difference was observed in TMPRSS2 protein abundance between sexes (Fig. 1i). The “housekeeping” protein α-tubulin was used as a loading and blotting control, with no variation observed in α-tubulin protein levels between groups (Supp.Fig. 4.a-d**)**. Non-cropped versions of full blots are shown in Supplemental Figs. 2 and 3. To further evaluate kidney cortex ACE2 in a more quantitative way, the protein concentration of ACE2 was measured by ELISA in kidney cortex homogenates from male and female patients above 80 yr. There was no significant difference between ACE2 protein normalized to the total protein concentration (Fig. 1j). Thus, ACE2 and TMPRSS2 protein levels in kidney cortex are not elevated with high age in males compared with females.

### ACE2 and TMPRSS2 protein in uEVs from healthy controls and SARS-CoV-2-infected patients

**Fig. 2:**
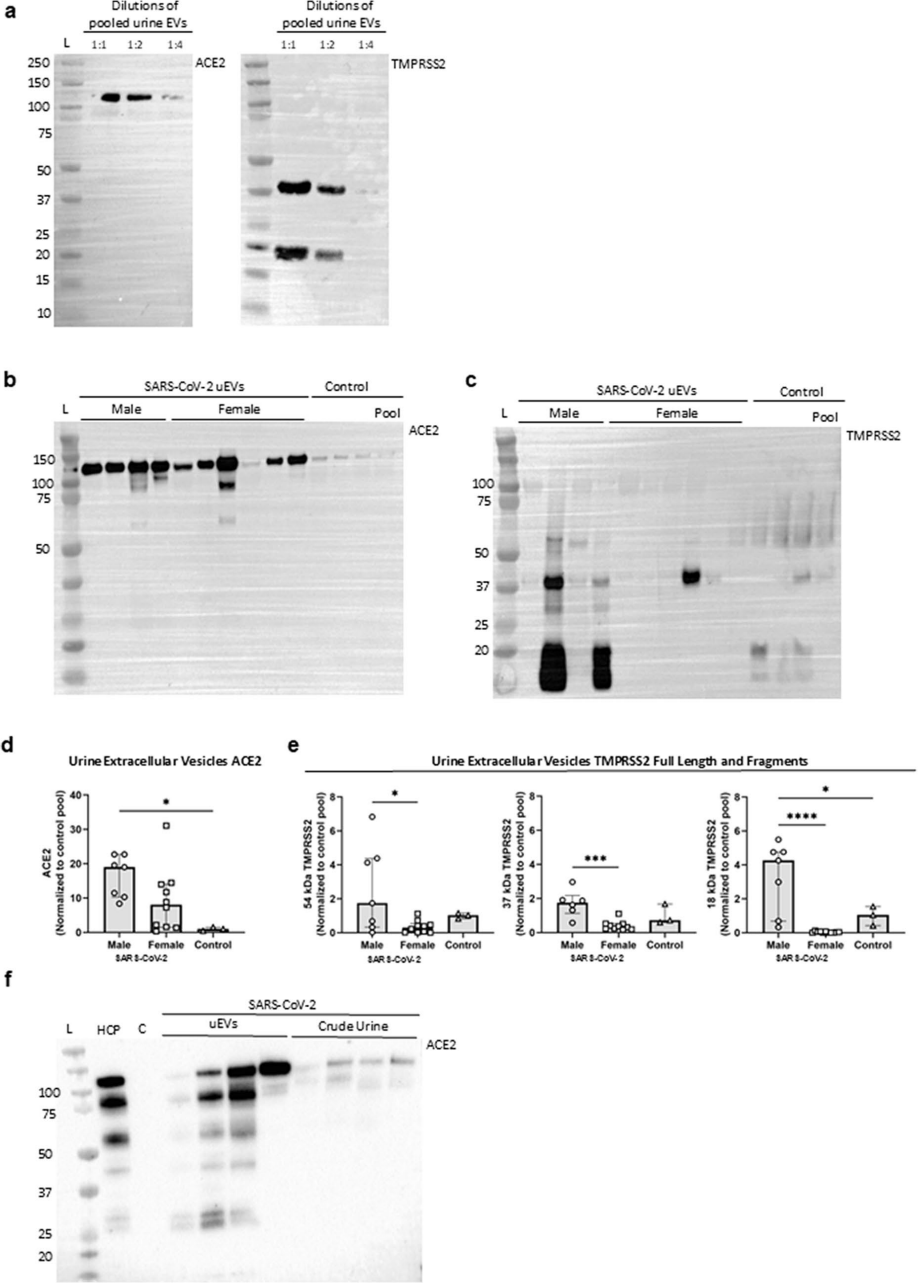
Detection of ACE2 and TMPRSS2 proteins in urine extracellular vesicles (uEVs) isolated from patients admitted to hospital with SARS-CoV-2 infection and from healthy control. **a** Immunoblot of pooled uEVs from healthy control persons for ACE2. Dilutions ranging from 1:1, 1:2, and 1:4. A single protein band was apparent with antibody directed against ACE2 at ~ 120 kDa. 2 bands reacted for TMPRSS2 migrating at ~ 37 kDa and ~ 18 kDa. **b–c** Immunoblotting analysis of uEVs isolated from patients with SARS-CoV-2 infection and healthy individuals (control). A protein band reacted with anti-ACE2 at ~ 120 kDa and for TMPRSS2, a protein presented at the predicted full-length of ~ 54 kDa, shorter, likely cleaved fragments at ~ 37 and ~ 18 kDa with polyclonal anti-TMPRSS2. **d** ACE2 protein abundance normalized to the control pool, and semi-quantification showed that the protein abundance of uEV-associated ACE2 was not different between sex but was significantly higher in males compared to the control (*p* = 0.03). **e** Full length and fragments of TMPRSS2 protein were normalized to the control pool. Semi-quantification showed a greater abundance of TMPRSS2 protein at ~ 54, ~ 37, and ~ 18 kDa in male patients than female (*p* < 0.05). The uEV protein abundance of TMPRSS2 fragment ~ 18 kDa was significantly greater in SARS-CoV-2-infected male compared to control. **f** Comparison of ACE2 protein abundance in uEVs versus crude urine from the same patients with SARS-CoV-2 by immunoblotting analysis. ACE2 was enriched in uEVs. Each bar represents the mean with ± SEM. *p* < 0.05 was considered significantly different. The number of asterisks reflects the degree of significance. Male *n* = 7, female *n* = 11, and *n* = 3 healthy control. L, ladder; HCP, human cortex pool; C, control urine

Urine extracellular vesicles served as a non-invasive “liquid biopsy” for assessing renal epithelial apical protein size and abundance. Serial dilution of precipitate by urine creatinine concentration (4 to 18 mmol/L) revealed significant detection of both proteins in EVs (Fig. 2a). ACE2 migrated at 120 kDa corresponding to glycosylated full-length protein (Fig. 2a). By contrast, EV-associated TMPRSS2 migrated at 37 kDa and 18 kDa, representing cleaved and/or degraded fragments. Notably, full-length TMPRSS2 at 54 kDa was not detected in uEV fractions (Fig. 2a). To examine if male patients with SARS-CoV-2 infection have more membrane-bound or shedded ACE2, immunoblotting was performed on uEVs isolated from a cohort of COVID-19 patients (> 60 yr), and from a cohort of young healthy males from a previous study [87]. Urine samples were collected on average 10.3 (± 2.3) days after symptom debut, 3.8 (± 1.6) days after admission, and 6.8 (± 3.6) days after COVID-19 diagnosis in patients (total n = 15) with moderate to severe disease admitted to a single center (for further patient characteristics see supp.Table 3). Samples with a Ucr < 4 µg/mL were not further investigated, leading to a drop in sample size (female *n* = 11, male *n* = 7, and healthy *n* = 3). ACE2 protein was detected at full length (~ 120 kDa) in all samples from patients with COVID (Fig. 2b). The abundance of ACE2 protein in uEVs from male patients was ~ 15 times higher than ACE2 in uEVs from healthy control males (*p* = 0.031) where a vague band of similar size was apparent (Fig. 2d). In some individual uEVs, a second, faster migrating, band appeared at ~ 85 kDa. This was inconsistent but appeared in 5 of 18 (28%) samples. Densitometry values normalized to healthy control pool showed no significant difference in ACE2 protein levels between male and female patients (*p* = 0.25). To reveal if there is enrichment of ACE2 in membranes, crude urine was compared with uEVs from the same patients. Normalizing to Ucr and subjecting the samples to immunoblot analysis showed that ACE2 is present at detectable levels also in crude urine and migrating as N-glycosylated full length (120 kDa) and truncated fragment (85 kDa), but with significantly greater abundance in uEVs (Fig. 2f).

Next, probing of uEVs from male patients with COVID-19 for TMPRSS2 revealed three distinct protein bands at 54 kDa, 37 kDa, and 18 kDa with the 54 kDa being the weakest. uEVs from female patients showed weak signal at 37 kDa or no signal, while uEVs from healthy males showed weak bands for TMPRSS2 at all three molecular sizes (Fig. 2c). Densitometric analysis showed significantly higher levels of TMPRSS2 protein in uEVs from male patients infected with SARS-CoV-2 compared to female patients for all moieties: full length and fragments at 54, 37, and 18 kDa (*p* < 0.05). The protein abundance of 18 kDa TMPRSS2 fragment was significantly elevated in males compared to non-infected control (*p* = 0.039) (Fig. 2e). Immunoblotting for aquaporin-1 (AQP1) was used as a positive membrane identification for uEVs from proximal tubular cells (Supp.Fig. 6). In summary, successful isolation of uEVs by PEG-precipitation identified intact ACE2 and cleaved TMPRSS2 protein; while there were no sex-difference in ACE2 in uEVs as in tissue, TMPRSS2 protein was more abundant in uEVs from male patients with COVID-19 compared to healthy control males and female patients with COVID-19, respectively, in contrast to kidney tissue.

### Co-localization of ACE2 and TMPRSS2 protein in uEVs from healthy controls and patients infected with SARS-CoV-2

**Fig. 3:**
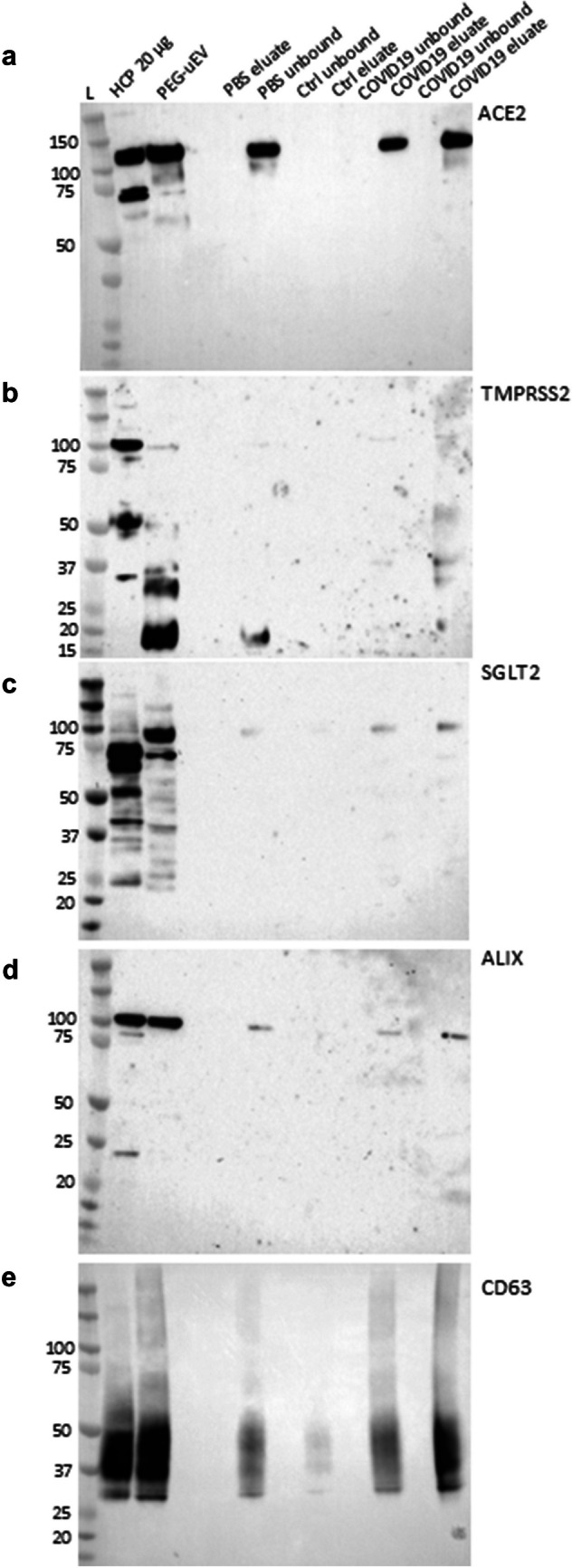
ACE2 and TMPPRSS2 do not co-localize in urine extracellular vesicles (uEVs) from patients with COVID-19. Poly-ethylene glycol (PEG)-precipitated uEVs from patients with COVID-19 and healthy control were subjected to secondary isolation by immunoaffinity purification for ACE2 with beads coated with an anti-ACE2 capture antibody. SDS-PAGE separation with subsequent immunoblotting for ACE2-immunoprecipitated proteins from uEVs allowed to study the potential co-isolation and thus co-clustering of protease TMPRSS2. 20 µg of human renal cortex tissue pool (HCP) and 10 µl of PEG-precipitated non-immunoprecipitated uEVs (PEG-uEV) served as positive control. Isolated uEVs incubated with PBS served as a negative control. uEV fractions from patients with COVID-19 captured by immunoaffinity for ACE2 (eluate) were positive for Sodium-glucose cotransporter-2 (SGLT2), CD63, and ALIX, but not TMPRSS2 neither full-length nor cleaved fragments. uEVs isolated from healthy control (Ctrl) were negative for ACE2 and TMPRSS2, but positive for CD63. The PBS unbound fraction was positive for uEVs while the PBS eluate (E) was negative. Pre-hoc predicted migration patterns were **a** 120 kDa for ACE2;** b** 54, 37, and 18 kDa for TMPRSS2; **c** 75 kDa for SGLT2; **d** 110 kDa for ALIX; and **e** 37–50 kDa for CD63. L, ladder

Since immunohistochemical labeling showed weak co-staining in cortex for ACE2 and TMPRSS2, the putative co-localization in cortex was studied by alternative method of co-immunoprecipitation using protein from uEVs. PEG-precipitated uEVs were subjected to immunoaffinity isolation with anti-ACE2 (#SN0754, Novus Biologicals®). ACE2 was detected in the evaluated fractions from patients with COVID-19, but not from the control (Fig. 3a). TMPRSS2 was not, or barely, detected in the ACE2-precipitated eluate of COVID-19 patients (Fig. 3b), suggesting no co-localization with ACE2 in uEVs. ALIX and CD63, internal and external EV markers respectively, were present in the evaluated fractions of the control and COVID-19 samples (Fig. 3d, e). Sodium-glucose cotransporter-2 (SGLT2), a proximal tubule marker, was present in the evaluated fraction but not in the unbound fraction from patients with COVID-19 (Fig. 3c). While the fraction of SGLT2-positive uEVs in the total uEV pool is not known, this indicates shedding of ACE2 from proximal tubular cells into the urine. PEG-precipitated uEVs incubated with PBS served as negative control, and PEG-precipitated uEV input and human cortex pool (HCP) served as positive controls.

### ACE2 urine abundance in relation to urine albumin, SARS-CoV-2 nucleoprotein, and disease severity

**Fig. 4:**
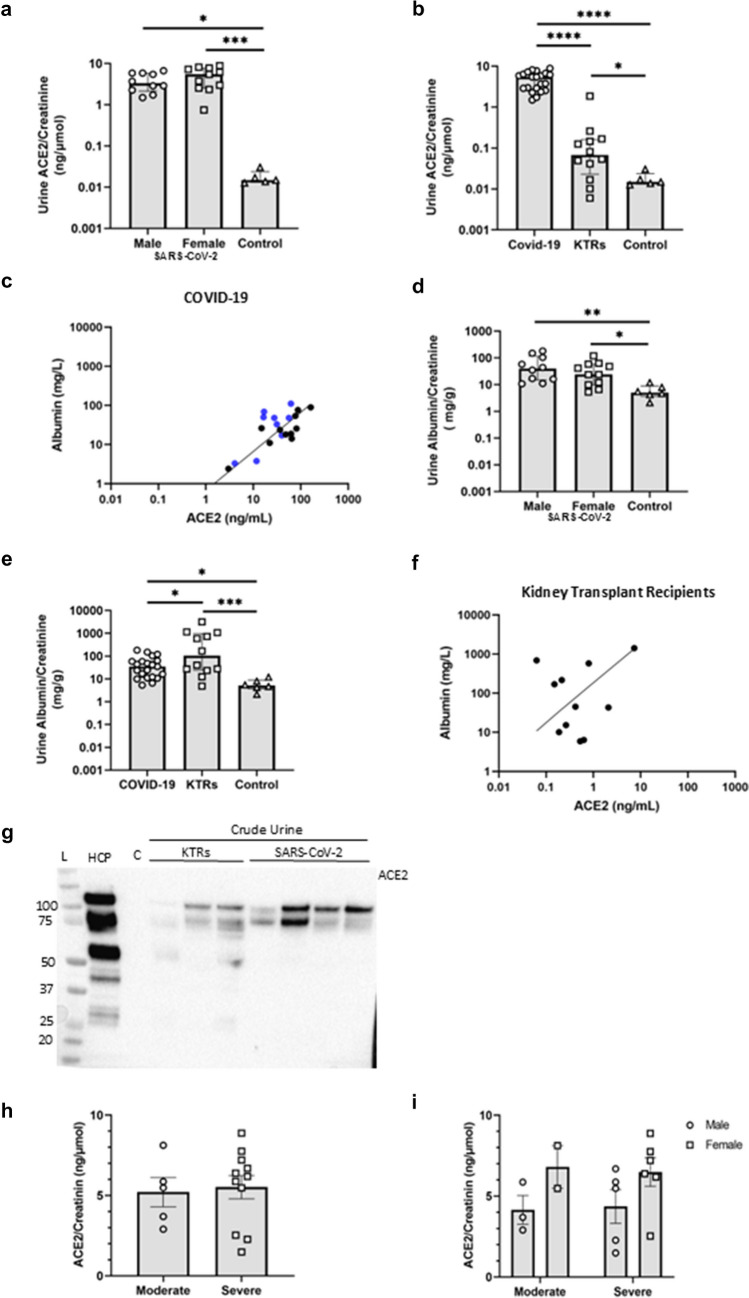
Urinary ACE2 and albumin excretion in patients with COVID-19, in patients with albuminuria without COVID-19 (kidney transplant recipients (KTRs)) and in healthy male controls. **a** Urine ACE2-to-creatinine ratio (ng/µmol) was significantly elevated (~ 200 times) in both male and female patients with COVID-19 infection compared to non-infected healthy controls (*p* < 0.05). **b** Patients with COVID-19 had a ~ 200-times greater urine ACE2/creatinine (ng/µmol) compared to KTRs and healthy controls (p < 0.0001). KTRs had a greater urine ACE2-to-creatinine ratio (ng/µmol) than healthy control persons (*p* = 0.03). **c** There was a significant direct relation (*R*^2^ = 0.7168, *p* = 0.0004) between urine ACE2 (ng/mL) and albumin (mg/L) in patients with COVID-19 infection (males plotted with blue dots, females shown as black dots). **d** There was no significant difference in urine albumin/creatinine (UACR) (mg/g) between male and female patients infected with SARS-CoV-2 but significantly higher excretion compared to healthy control persons (*p* < 0.05). **e** KTRs had significantly elevated UACR (mg/g) compared to patients with COVID-19 and healthy controls (*p* < 0.05) and patients with COVID-19 had higher UACR (mg/g) compared to healthy controls (*p* = 0.0299). Data were log-transformed to yield normal distribution and plotted on a log scale as median ± interquartile range. **f** In KTRs, urine ACE2 and albumin concentrations did not relate significantly (*R*^2^ = 0.1502, *p* = ns). The data were log-transformed to yield normal distribution and plotted on a double logarithmic scale. **g** ACE2 immunoblotting of crude urine samples from KTRs vs patients with COVID. C, control urine. Variable volumes of urine with same absolute amount of creatinine were loaded and separated by SDS-PAGE. The predicted migration of full length ACE2 is ~ 120 kDa. **h** Urine ACE2 protein levels normalized to creatinine (ng/µmol) did not correlate with COVID-19 disease severity (*R*.^2^ = 0.066, *p* = 0.807). Similarly, when split into sex** i** urine ACE2 protein levels did not differ depending disease severity. P < 0.05 was considered statistically significant, and the number of * shows the degree of significance. Male patients with COVID-19 *n* = 7, female patients *n* = 11, non-infected healthy controls *n* = 6, and KTRs *n* = 12

Crude spot urine samples from SARS-CoV-2 patients and healthy controls were quantitatively analyzed for albumin and ACE2 by ELISA and normalized to urine creatinine concentration**.** ACE2 urine levels were significantly higher in patients with SARS-CoV-2 compared to healthy male controls ($\sim$ 217 and $\sim$ 376 times), with no difference between male and female patients (Fig. 4a). Urine-albumin creatinine-ratio (UACR) was elevated in patients with COVID-19 compared to healthy controls (males 8 times, females 4.5 times) with no sex-based difference (Fig. 4d). There was a positive and significant direct relation between urine ACE2 (ng/mL) and urine albumin (mg/L) in patients with COVID-19 (Fig. 4c, *p* = 0.004); this was similar for both sexes (male (*r*^2^ = 0.7873, *p* = 0.069) and female (*r*^2^ = 0.8540, *p* = 0.0004), Supp.Fig. 7). To ascertain the degree of aberrant filtration of ACE2 versus shedding, urine samples were analyzed from patients with albuminuria but without COVID-19 (kidney transplant recipients, *n* = 12) from a previous study [33]. KTRs excreted significantly lower amounts of ACE2 compared to COVID-19 patients (*p* < 0.0001) but higher than healthy controls (13 times, Fig. 4b). Immunoblotting of crude urine revealed a greater abundance of ACE2 immunoreactive bands in patients with COVID-19 compared to KTRs, both full length (120 kDa) and truncated (85 kDa) (Fig. 4g). KTRs exhibited significantly, 12 times, higher UACR compared to patients with COVID-19 and 100 times higher than healthy controls (6 ± 3.4 mg/g) (*p* < 0.05) (Fig. 4e). However, urine ACE2 and albumin did not significantly correlate in KTRs (Fig. 4f) (*r*^2^ = 0.1502, *p* > 0.05). Thus, albuminuria cannot account fully for increased urine ACE2 in patients with COVID-19.

Further, a point-biserial correlation was performed to evaluate the relationship between COVID-19 disease severity and urine ACE2 normalized to creatinine. Severity of COVID-19 disease, a categorical variable with two levels: moderate and severe, was converted to a binary numeric variable (moderate = 0, severe = 1). The analysis yielded a correlation coefficient of 0.066 (*p* = 0.807), with a 95% confidence interval ranging from − 0.444 to 0.5444 (Fig. 4h). These results indicate a very weak and statistically non-significant association between COVID-19 disease severity and urine ACE2 levels. To estimate the effect of sex and disease severity as predictors of urine ACE2 levels, a multiple linear regression was performed. Sex (male vs female) and disease severity (moderate vs severe) is considered independent variables, and urine ACE2 (ng/µmol creatinine) is considered the dependent variable. The regression model explains that 28.02% of the variability in urine ACE2 levels can be attributed to disease severity and sex (*R*^2^ = 0.2802). However, the overall fit of the model did not reach statistical significance (F(2, 13) = 2.531, p = 0.118), suggesting that disease severity and sex combined do not affect urine ACE2 levels (Fig. 4i).

Detection of SARS-CoV-2 nucleoprotein in urine samples of patients with COVID-19 was below detection limit (< 8.9 ng/L), and PCR analysis for SARS-CoV-2 transcripts; nucleocapsid (N), Envelope (E), Sub-Orf7A, L452R, and Sub-E yielded negative results. Similarly, isolated uEVs from SARS-CoV-2 patients showed no detectable SARS-CoV-2 N-protein and transcripts (Table 1). In summary, COVID-19 patients, regardless of sex and disease severity, have increased loss of ACE2 into urine, which clinically significant degree of albuminuria cannot account fully for, indicating, additional, shedding of ACE2. SARS-CoV-2-related protein and mRNA were undetected in urine and uEVs.

**Table 1:** Results of analyses of urine and urine extracellular vesicles for SARS-CoV-2 Nucleoprotein and RT-qPCR analysis of nucleocapsid (N)-gene, L452R mutation, Envelope (E)-gene, Sub-Orf7A, and Sub-E, which were either below detection limit (< 8.9 ng/L) or negative

Characteristics	Total (*n* = 22)
Male/female	10/12
Age years	> 60 yr
Urine	
SARS-CoV-2 Ag (ng/L)	< 8.92
N-gen	NEG
L452R	NEG
E-gen	NEG
Sub-Orf7A	NEG
Sub-E	NEG
Urine extracellular vesicles	
SARS CoV-2 Ag (ng/L)	< 8.92

## Discussion

The present study shows significant ACE2 and TMPRSS2 protein in human kidney cortex in both male and female individuals. While levels were not different within age groups below 50 yr vs above 75 yr, females displayed higher ACE2 across the whole population (age 35–88 yr). Cortex tissue from males and females > 80 yr showed no difference in ACE2 protein. Thus, the hypothesis that older males have higher ACE2 and TMPRSS2 protein in the kidney was refuted, at least for the age groups that were available for analysis (youngest male patient enrolled below 50 was 38 yr). Interestingly, isolated uEVs from male patients with COVID-19 admitted to the hospital presented greater protein abundance of ACE2 and TMPRSS2 compared to control. Consistent with the kidney tissue analysis, ACE2 and TMPRSS2 did not co-localize in uEVs from apical membranes. Urine ACE2 levels correlated with urine albumin levels in patients with COVID-19 but although patients had clinically significant albuminuria, the aberrant filtration could not account quantitatively for the increase in ACE2 as revealed in kidney transplant recipients with higher level of albuminuria but less ACE2. If ACE2 dramatically increases in plasma in patients with COVID concurrent with albuminuria, more ACE2 would be filtered. However, SARS-CoV-2 is likely to also promote shedding of proximal tubular ACE2 into the tubular fluid/urine although urine ACE2 did not correlate with COVID-19 disease severity and sex. No SARS-CoV-2 mRNA or nucleoprotein were detected in crude urine or uEVs from hospitalized patients with severe COVID-19. These negative findings from urine are similar to most other published data [25, 39, 60].

It is known that tissue ACE2 is influenced by sex [22, 36, 51]. Estrogen favors the expression of ACE2 compared to ACE [10], promoting signaling through the Ang1-7-MAS receptor axis and reduces the activation of the ANGII-AT1R arm of the renin-angiotensin-aldosterone system [9, 79, 90]. In the kidney tissue samples investigated from patients above 80 yr, females and males had similar level of ACE2 protein in the kidney cortex. Tissue differences in kidney ACE2 could be driven by the younger females. SARS-CoV-2 requires ACE2 and co-localization with TMPRSS2 is crucial for S-protein proteolytic processing and conformational changes that lead to membrane fusion in human lung epithelial cells [6, 24]. Therefore, TMPRSS2 levels and co-localization, rather than ACE2 abundance alone, may influence age- and sex-based susceptibility differences. However, adjacent human kidney sections revealed only minor overlap in certain cortex segments, with no overlap in the medulla. To our knowledge, TMPRSS2 protein has not been detected in human kidney tissue before by immunohistochemistry and immunoblotting. Bertram et al. showed TMPRSS2 protein in several human epithelial tissues with no data from kidney tissue [5]. *In situ* hybridization and RNA-sequencing reveal TMPRSS2 RNA in the distal convoluted tubule and collecting duct of human [75] and rodent kidney [12, 45] which would fit the present observation.

Immunoaffinity isolation of uEVs from COVID-19 patients supported the notion that there was no co-clustering of ACE2 with TMPRSS2 at apical membranes from kidney or urinary tract epithelia. These findings suggest that most kidney tubular cells may not be highly susceptible to the SARS-CoV-2 virus due to less abundant co-presentation of the necessary entry machinery compared to pneumocytes. However, other proteases besides TMPRSS2 could facilitate SARS-CoV-2 entry [6].

In line with a previous study of urine from COVID-19 patients [77], we show that SARS-CoV-2 patients have a higher degree of ACE2 protein in crude urine and in uEVs compared to healthy controls. Kidney tissue analysis was not feasible in infected patients. The contribution of aberrant filtration versus epithelial shedding of ACE2 in patients with COVID-19 was addressed by comparison with patients with albuminuria without COVID-19 (KTRs). Despite KTRs having a greater degree of albuminuria, they had markedly lower levels of ACE2 compared to patients with COVID-19. This suggests a significant increase in kidney epithelial cell shedding and/or EV release rate in COVID-19 patients. We found that human kidney ACE2 is significantly glycosylated, it appears dominantly at full length but with shorter truncated protein established as a shedded variant [53, 84]. In ANGII-mediated hypertensive mice, both shedded full-length and the soluble amino-terminal extracellular catalytic domains of ACE2 are detected in urine [53]. Kidney-specific knockout of ACE2 in mice confirms full-length protein shedding by a local mechanism since full-length ACE2 is eliminated in urine in such mice [53]. The dominant immunoreactive band detected in both crude urine and enriched in EVs from SARS-CoV-2 patients likely represents full-length membrane-attached ACE2 shed from the proximal tubule apical membrane. The lower size band may originate from ACE2 degradation in urine [84] or cleavage by ADAM17 [53]. Shedding can functionally relocate ACE2 activity and increase it in urine [29, 85]*.* Stressed cells have a higher rate of EV release [3, 50, 72]*,* and although data point to patients with COVID-19 shed more EVs into urine compared to healthy individuals, the mechanism is unknown. In summary, COVID-19 is associated with predominant shedding and loss of ACE2 from proximal tubular epithelial cells, potentially altering ANGII metabolism in the kidney.

The uEVs isolated from patients with SARS-CoV-2 and healthy control males showed TMPRSS2 protein corresponding to full-length zymogen (54 kDa) and cleaved fragments (37 and 18 kDa). To reach its active form, the TMPRSS2 zymogen undergoes proteolytic processing either post-translationally during synthesis or at the apical membrane [54, 74]. Several studies identified the mature catalytic chain of TMPRSS2 at 24–35 kDa dependent on the expression system [1, 71]. The physiological function of TMRPSS2 remains elusive, but it is known to be able to cleave and activate ENaC, and is highly sensitive to androgens testosterone and dihydrotestosterone (5-DHT), particularly in the prostate epithelium [1, 47, 49, 71]. uEVs from male patients with COVID-19 exhibited higher protein abundance of full-length and fragments compared to female patients. In nephrectomy cortex tissue there was no sex difference in TMPRSS2 protein abundance. By contrast, TMPRSS2 protein level in uEVs from patients with SARS-CoV-2 infection appears to be sex-dependent. This discrepancy could originate from differential regulation in the kidney medulla by androgens or be due to release from the prostate gland or seminal vesicles highly sensitive to 5-DHT.

The present cohort of COVID-19 patients was infected with the SARS-CoV-2 alpha variant. As SARS-CoV-2 mutates and new subvariants emerge, these variants have become significantly more responsible for the severity of disease than sex [11]. Indeed, the ability to infiltrate the kidney epithelium could be associated with the variable virus affinity for the ACE2 receptor [59, 86]. Several studies have reported cases of AKI in patients hospitalized with SARS-CoV-2 infection [34], while others have found AKI to be uncommon [81]. Multiorgan failure has been suggested as causative for AKI rather than SARS-CoV-2 renal tropism [15]. Post-mortem detection of SARS-CoV-2 in kidney tissue has been inconsistent [7, 60]: George et al. identified S-protein in urine samples from 23 out of 95 SARS-CoV-2 positive individuals [26], and Veyrenche et al. showed that nucleocapsid antigen in urine associated better with ICU admission than nucleocapsid antigen in plasma [78]. The present cohort of patients with COVID-19 displayed varying level of clinical albuminuria and thus circulating SARS-CoV-2 nucleoprotein (50 kDa) and other SARS-CoV-2-related proteins could potentially be filtered aberrantly in the active infection phase with plasma viral load. On average, the patients with COVID-19 had urine collected 10.3 ± 2.3 days from symptom debut, 6.8 ± 3.6 days from COVID-19 diagnosis, and 3.8 ± 1.6 days from hospital admission. At this stage of the infection, we did not detect SARS-CoV-2 mRNAs or protein in urine or uEVs. Multiple studies report low detection rate of SARS-CoV-2-related proteins and genes in urine [25], with a crude overall detection rate of 4.5% [39], consistent with our negative findings in both crude urine and uEVs. In COVID-19, AKI is more likely a consequence of systemic changes and potentially elevated local ANGII rather than direct SARS-CoV-2 infiltration of the renal epithelium [76]. Systemic loss of ACE2 in mice generates a higher ANG-II-mediated mean arterial blood pressure [53].

Urine ACE2 levels in the present cohort did not correlate with COVID-19 disease severity nor sex. One study relates SARS-CoV-2 N-protein in urine with AKI [73]. Vergara et al. relate urine ACE2 with tubular injury markers and AKI in severe COVID-19 disease [77]. Unilateral ischemia-reperfusion injury causes loss of ACE2 into urine correlating with loss of kidney ACE2 protein *in vivo* [66]. The present study did not clarify the cause for the shedding of ACE2 observed in response to SARS-CoV *in vitro* [27, 30]. It could be a compensatory mechanism to decrease susceptibility to infection or a side-effect of ADAM17 activation by systemic infection [40, 48]. ADAM17 activity is associated with the risk of cardiovascular events in CKD [55], and various “stressors,” such as high glucose and ANGII [23, 85] can increase ADAM17 in the kidney. Thus, shedding of ACE2 may not be a consequence of direct kidney infiltration, but more so a systemic response to infection.

In summary, our findings suggest that ACE2 and TMPRSS2 proteins are not more abundant in the human kidney cortex of males with high age and do not co-localize. In patients with severe COVID-19 admitted to hospital, urine ACE2 increase 2–300 times with no sex difference, while TMPRSS2 is more abundant in uEVs from male patients. SARS-CoV-2 antigen and mRNA are undetectable in urine and EVs.

## Conclusion

Higher kidney ACE2 and TMPRSS2 protein levels are unlikely to explain the higher susceptibility to SARS-CoV-2 in males and elderly. Kidney tubular cells appear not highly susceptible to SARS-CoV-2 infection. Loss of membrane-bound and plasma ACE2 into urine during SARS-CoV-2 infection may impact ANGII metabolism with consequences for systemic blood pressure and kidney function.

## Clinical perspectives

- SARS-CoV-2 virus engages ACE2 and requires TMPRSS2 activity. High age and male sex are risk factors for disease. Acute kidney injury is associated with SARS-CoV-2 infection. Whether it is systemic or a consequence of direct renal infiltration is controversial.
- ACE2 and TMPRSS2 protein levels in human kidney cortex are not higher in males than females and with high age, and the two proteins do not cluster. ACE2 in urine and uEVs from COVID-19 patients, regardless of sex, is highly elevated which clinical albuminuria cannot account fully for. TMPRSS2 is higher in male uEVs. SARS-CoV-2-related protein, mRNA, and antigen are undetectable in urine and uEVs.
- A difference in kidney ACE2 protein is less likely to account for higher susceptibility to SARS-COV-2 in males or elderly. Loss of membrane-bound kidney ACE2 and circulating ACE2 into urine could impact (lower) ANGII metabolism to vasoprotective Ang1-7 and have negative systemic consequences for blood pressure and long-term organ fibrosis.

## Supplementary information

Below is the link to the electronic supplementary material.

Supplementary file1 (PDF 800 KB)

## Data Availability

All data generated as part of this study are available from the corresponding author upon reasonable request.

## Abbreviations

ACE: Angiotensin-converting enzyme
ADAM17: A disintegrin and metalloprotease 17
ALIX: ALG-2-interacting protein X
AKI: Acute kidney injury
ANGII: Angiotensin II
ANOVA: Analysis of variance
AQP1: Aquaporin 1
CD63: Cluster of differentiation 63
CKD: Chronic kidney disease
COVID-19: Coronavirus disease-19
DAB: 3,3-Diaminobenzidine
DM: Diabetes mellitus
5-DHT: Dihydrotestosterone
E gene: Envelope gene
ELISA: Enzyme-linked immunosorbent assay
HCP: Human cortex pool
ICU: Intensive care unit
KO: Knockout
KTR: Kidney transplant recipient
LFIA: Lateral Flow Immunoassay
N-gene: Nucleocapsid gene
N-protein: Nucleoprotein
ON: Overnight
PBS: Phosphate-buffered saline
PNGase F: N-Glycosidase F
PEG: Polyethylene glycol
RT: Room temperature
SARS-CoV-2: Severe acute respiratory syndrome corona virus 2
SEM: Standard error of mean
SGLT2: Sodium-glucose cotransporter-2
S-protein: Spike protein
Strep-HRP: Streptavidin-conjugated horseradish peroxidase
sgRNA: Subgenomic RNA
TMB: 3,3′,5,′-Tetramethylbenzidine
TMPRSS2: Transmembrane protease, serine 2
uEVs: Urine extracellular vesicles
UACR: Urine albumin-to-creatinine ratio
