# Supplementary material for: ACE2 and TMPRSS2 in human kidney tissue and urine extracellular vesicles with age, sex, and COVID-19
Source: Pflugers Arch. 2024 Oct 9;477(1):83–98. doi: 10.1007/s00424-024-03022-y (PMC11711140; doi:10.1007/s00424-024-03022-y)
Supplement: Supplementary file 1 — Supplementary file1 (PDF 800 KB) [file 424_2024_3022_MOESM1_ESM.pdf]

Supplemental Table 1

Male				Female			
Ref. No.	< 50 years	Ref. No.	> 75 years	Ref. No.	< 50 years	Ref. No.	> 75 years
1	47 years	7	77 years	A	48 years	G	78 years
2	48 years	8	78 years	B	39 years	H	78 years
3	48 years	9	82 years	C	48 years	I	78 years
4	44 years	10	88 years	D	38 years	J	82 years
5	38 years	11	78 years	E	41 years	K	78 years
6	44 years	12	81 years	F	35 years	L	86 years

Supplemental table 1: Age and sex from 24 patients that underwent nephrectomy and where kidney cortex tissue was collected from the biobank. Each cortex homogenate sample from male was given a randomized number and samples from female were given a letter.

Supplemental Table 2

Male >60 years					
Ref. No.	Disease Severity	Date of Admission	Date of COVID-19 diagnosis	Date of symptom debut	Date of Urine collection
101	Moderate	2020-12-14	2020-12-11	2020-12-05	2020-12-18
102	Moderate	2020-12-14	2020-12-07	2020-12-06	2020-12-18
103	Severe	2020-12-11	2020-12-06	2020-12-08	2020-12-16
106	Severe	2020-12-16	2020-12-11	2020-12-09	2020-12-18
107	Severe	2021-01-02	2021-01-02	2020-12-26	2021-01-04
109	Severe	2020-12-29	2020-12-29	2020-12-29	2021-01-04
114	Severe	2021-01-09	2021-01-01	2021-01-01	2021-01-01
118	Moderate	2021-01-19	2021-01-19	2021-01-10	2021-01-22
125	N/A	2021-02-24	2021-02-24	2021-02-24	2021-02-23
126	N/A	2021-03-05	N/A	N/A	N/A

Female >60 years					
Ref. No.	Disease Severity	Date of Admission	Date of COVID-19 diagnosis	Date of symptom debut	Date of Urine collection
104	Moderate	2020-12-10	2020-12-04	2020-12-07	2020-12-16
105	Severe	2020-12-15	2020-12-16	2020-12-05	2020-12-18
110	Severe	2021-01-04	2020-12-28	02-01-2021	N/A
111	Severe	2021-01-04	2020-12-30	2021-01-02	2021-01-08
112	Severe	N/A	2020-12-29	N/A	2021-01-08
115	Moderate	2021-01-18	2021-01-08	2021-01-05	2021-01-12
116	Severe	2021-01-17	2021-01-09	2021-01-04	2021-01-18
120	Severe	2021-01-25	2021-01-20	2021-01-12	2021-01-12
121	N/A	N/A	N/A	N/A	N/A
122	N/A	N/A	N/A	N/A	N/A
123	N/A	N/A	N/A	N/A	N/A
124	N/A	N/A	N/A	N/A	N/A

Supplemental Table 2: Characteristics of patients with COVID-19 including disease severity, dates of admission, covid-19 diagnosis, symptom debut and urine collection .

Supplemental Table 3

	No. Of patients	Average time from symptom debut to urine collection (days)	Average time from covid-19 diagnosis to urine collection (days)	Average time from admission to urine collection (days)
All patients	15	10.3 (±2.3)	6.8 (±3.6)	3.8 (±1.6)
Female	6	10.8 (±2.6)	8.2 (±4)	3.8 (±1.6)
Male	9	9.9 (± 1.9)	5.9 (±3.1)	3.8 (±1.5)

Supplemental Table 3: Average time from debut of symptoms, to COVID-19 diagnosis, and time from hospital admission to urine collection (days).

Supplemental Table 4

Primary AB	Dilution	Incubation	Secondary AB	Dilution	Predicted molecular weight/kDa	Ab Solvent
**TMPRSS2 (Cat. No. HPA035787, Sigma Polyclonal)**	1:2000	4°C ON	HRP-goat-anti-rabbit	1:2000	54	TBST
**ACE2 (Cat. No.SN0754, Novus Biologicals®)**	1:2000	4°C ON	HRP-goat anti-rabbit	1:2000	120	TBST
**AQ1 (B-11, Cat. No. Sc-25287, Santa Cruz Biotechnology)**	1:1000	4°C ON	HRP-goant anti-rabbit	1:2000	24	TBST
**ALIX (3A9, Cat. No. Sc-53538, Santa Cruz Biotechnology)**	1:1000	4°C ON	HRP-goat anti mouse	1:2000	110	TBST
**Sodium-Glucose Transporter 2 (D-6, Cat. No. Sc-393350, Santa Cruz Biotechnology)**	1:1000	4°C On	HRP-goat anti mouse	1:2000	70	TBST
**CD63 (Ca. No. Sc-5275, Santa Cruz Biotechnology)**	1:1000	4°C ON	HRP-goat anti mouse	1:2000	50-37	TBST

Supplemental Table 4: Applied antibodies for immunoblotting and immunohistochemistry

Supplementary Figure 1

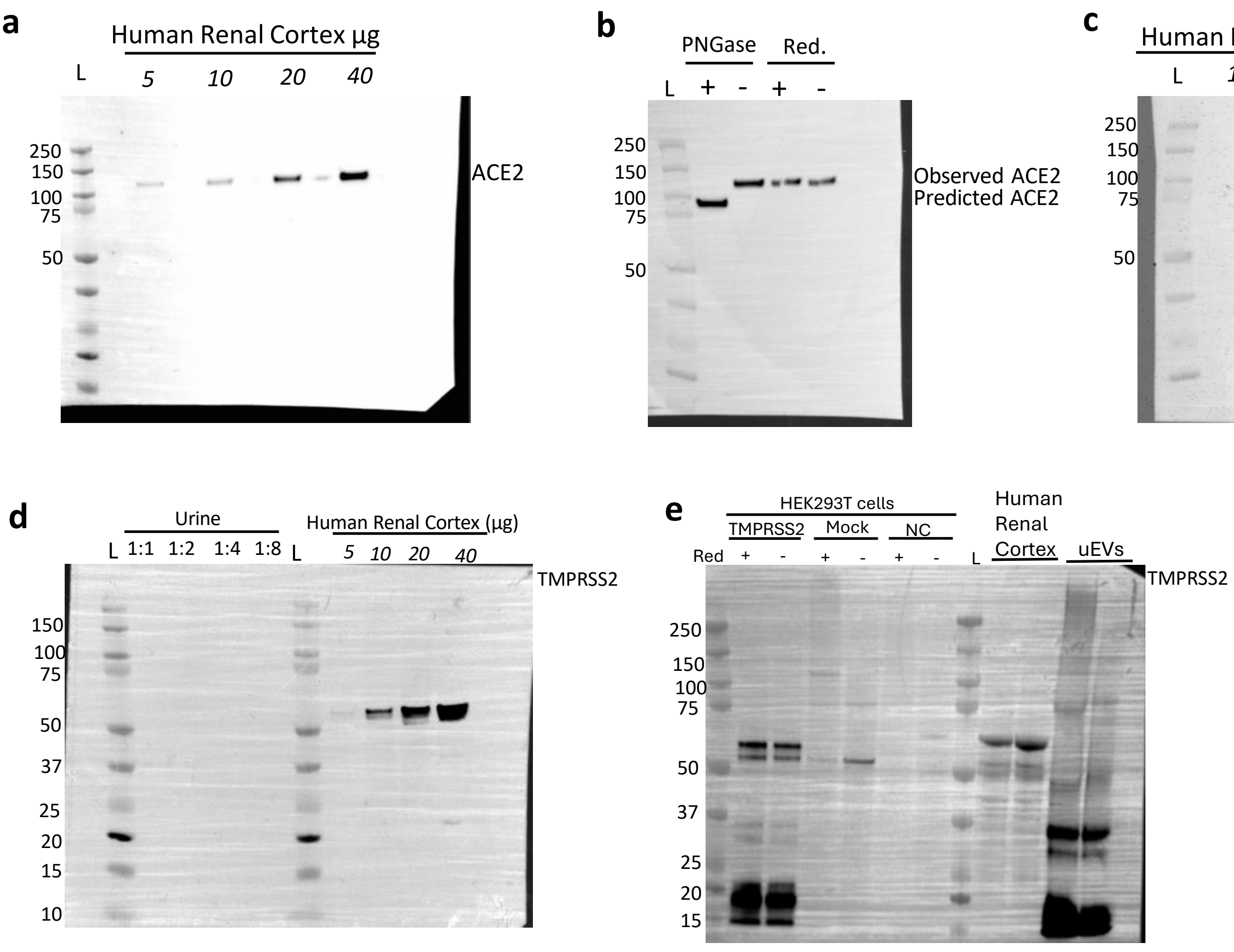

Supplementary figure 1: **a)** Immunoblotting for ACE2 protein with human renal cortex homogenate pool using increasing total protein: 5, 10, 20 and 40 µg. A protein was detected at ~120 kDa **b) I**mmunoblotting for ACE2 with or without deglycosylation by PNGase and reducing agent (Red.). PNGase treatment introduced a shift ~92 kDa compatible with predicted weight of ACE2 **c)** Negative control. Immunoblot of cortex pool incubated only with secondary antibody. **d)** Immunoblotting for TMPRSS2 protein with human renal cortex pool and urine. Urine was diluted 1:1, 1:2, 1:4, and 1:8 with no signal. With increasing amounts of homogenate 5, 10, 20 and 40 µg a single band was detected at ~54 kDa compatible with TMPRSS2. **e)** Immunoblotting for TMPRSS2 in human renal cortex, urine extracellular vesicles (uEVs), HEK293T cells transfected with TMPRSS2(#NBL1-17121, 1µg/µl), mock HEK293T cell lysate, and negative control (NC) cell lysate with or without reducing agent (+/-). TMPRSS2 protein, zymogen and cleaved fragments were detected at 54, 50, 37, 30, and 18 kDa in TMPRSS2 overexpressing cells. HEK293T mock cell lysate revealed a band at 50 kDa, corresponding to a band in TMPRSS2 overexpressing cells. Negative cell lysate showed no signal for TMPRSS2. L, ladder. Red., Reducing agent

Supplementary Figure 2

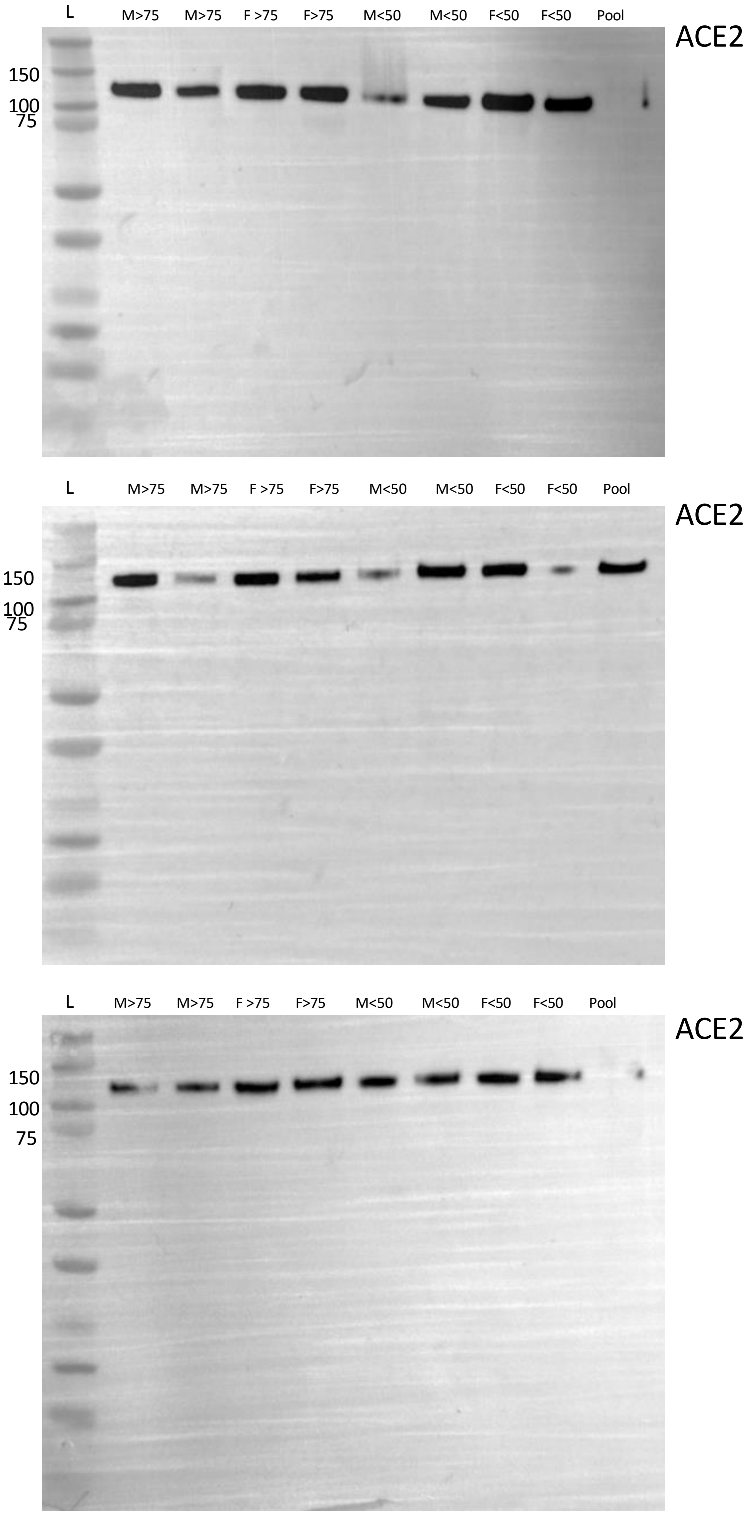

Supplementary figure 2: Detection of ACE2 protein in human renal cortex by immunoblot analysis. Lanes 1-2: Male >75, lanes 3-4: Female>75, lanes 5-6: Male<50, lanes 7-8: Female<50 and lane 9: positive control pool of human kidney cortex. ACE2 presented at $\sim$120 kDa in lane 1-9. n=6 for male>75, n=6 for female>75, n=6 for male<50 and n=6 for female<50.

Supplementary Figure 3

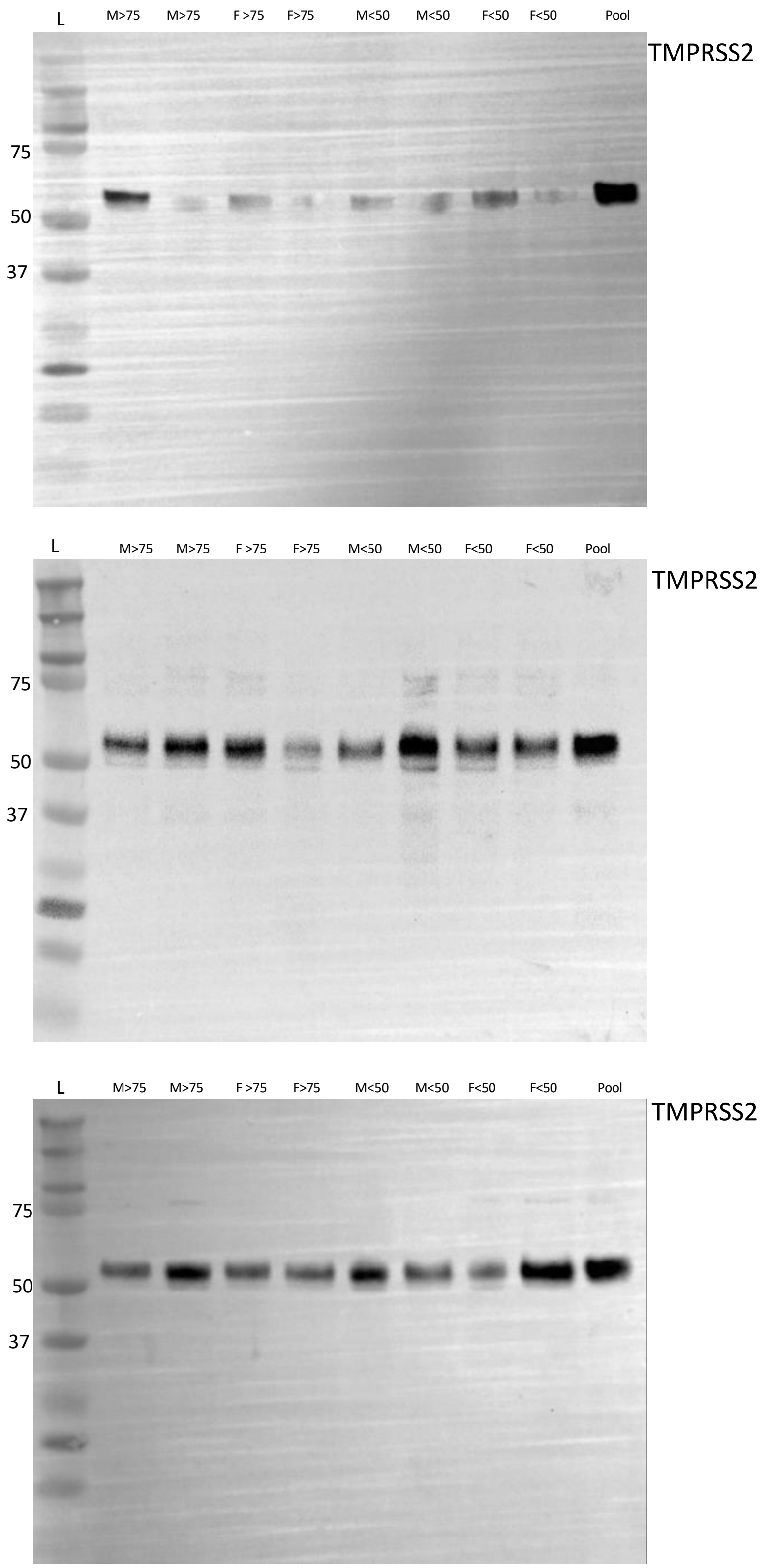

Supplementary figure 3: Immunoblotting with anti-TMPRSS2 antibody using human renal cortex homogenate. Lanes 1-2: Male >75, lanes 3-4: Female>75, lanes 5-6: Male<50, lanes 7-8: Female<50 and lane 9: positive control pool of human kidney cortex. TMPRSS2 presented at ~54 kDa. n=6 for male>75, n=6 for female>75, n=6 for male<50 and n=6 for female<50.

Supplementary Figure 4

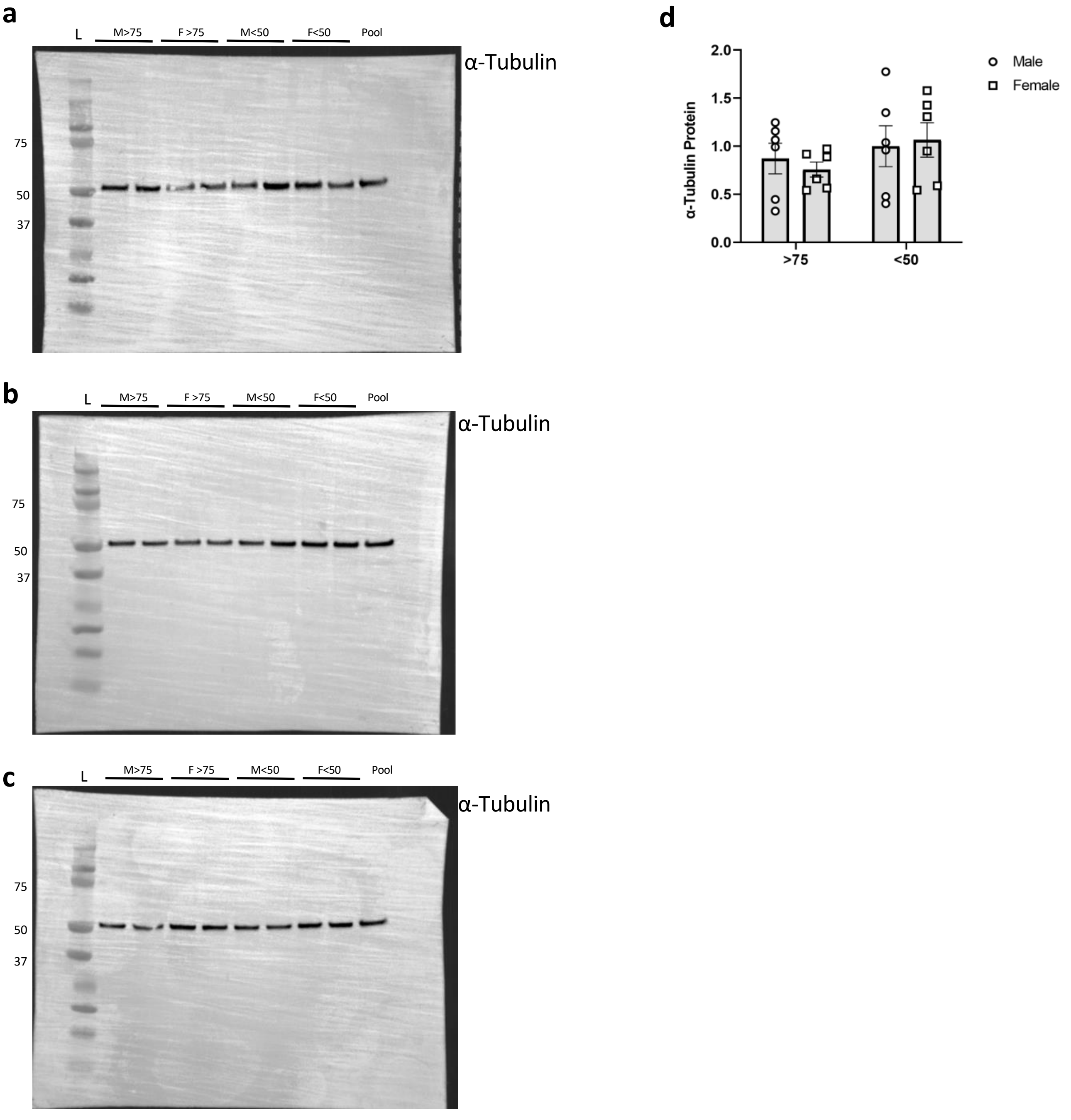

Supplementary figure 4: **a-c)** Detection of α-Tubulin protein in human renal cortex by western blotting analysis. Lanes 1-2: Male >75, lanes 3-4: Female>75, lanes 5-6: Male<50, lanes 7-8: Female<50 and lane 9: positive control pool of human kidney cortex. α-Tubulin presented at ~50 kDa in lane 1-9. n=6 for male>75, n=6 for female>75, n=6 for male<50 and n=6 for female<50. **d)** α-tubulin protein abundance normalized to the average of <50 y male signal, and semi-quantification showed similar protein abundance in all samples.

Supplementary Figure 5

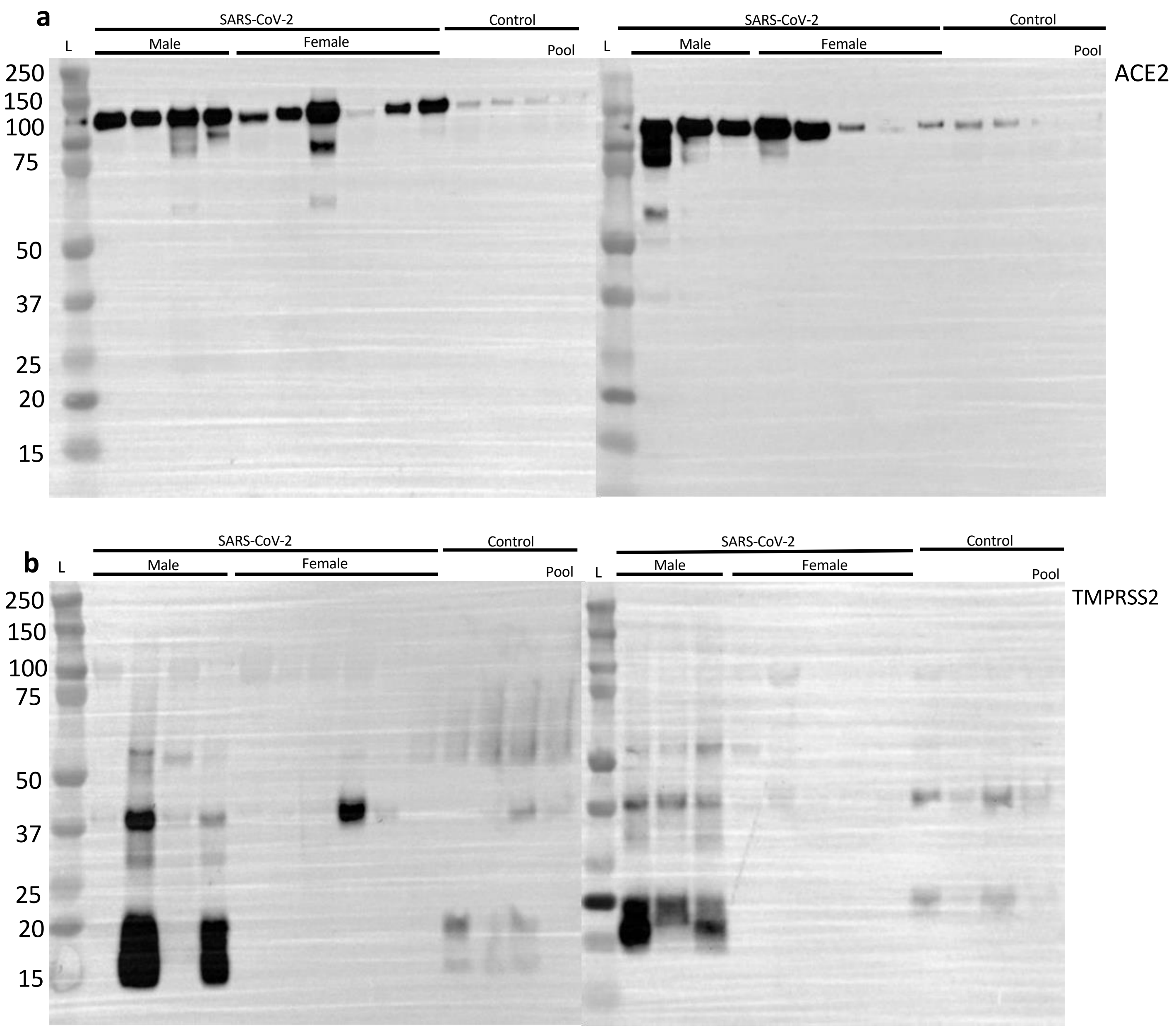

Supplementary figure 5: Non-cropped immunoblots using antibodies directed against **a)** ACE2 and **b)** TMPRSS2 using urine extracellular vesicles from patients infected with SARS-CoV-2 (male n=7, female n=11), and healthy controls (n=3). ACE2 protein detected at 120 kDa and TMPRSS2 protein at full length (54 kDa) and cleaved fragments (37 and 18 kDa).

Supplementary Figure 6

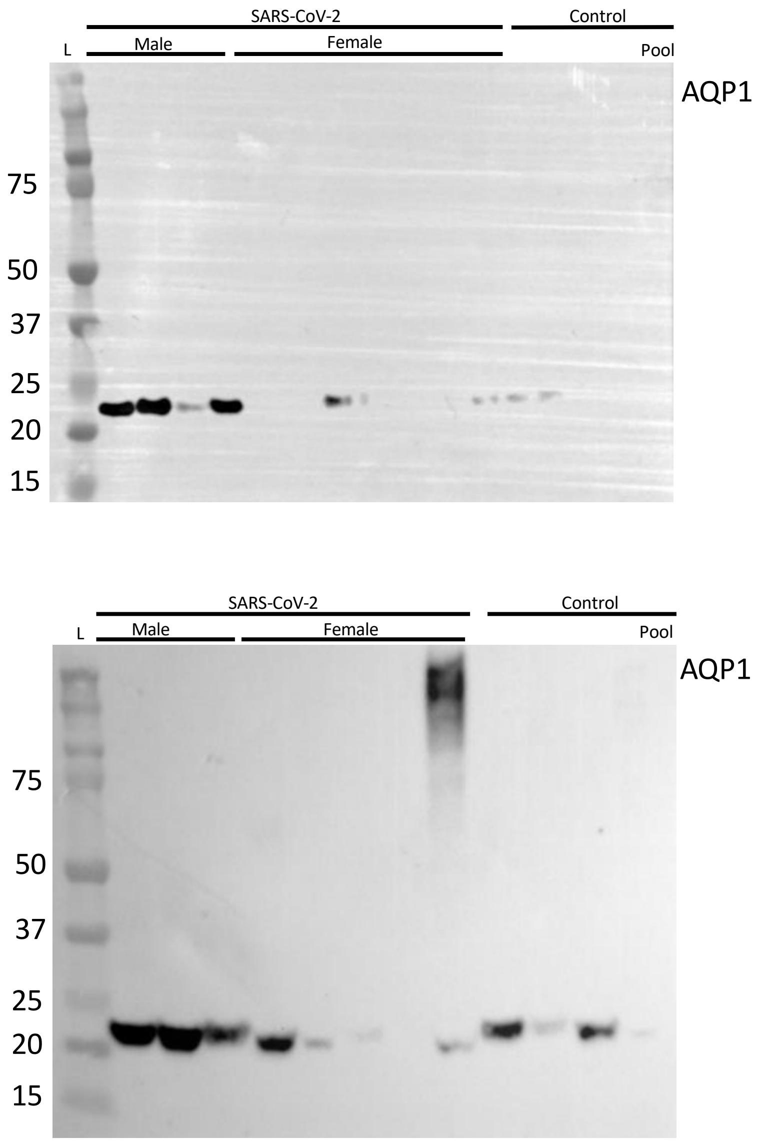

Supplementary figure 6: Non-cropped immunoblot detection of Aquaporin 1 (AQP1) protein abundance in urine microveiscles from patients infected with COVID-19 (male n=7, women n=11), and healthy controls (n=3). AQP1 protein was detected at ~21 kDa.

Supplementary Figure 7

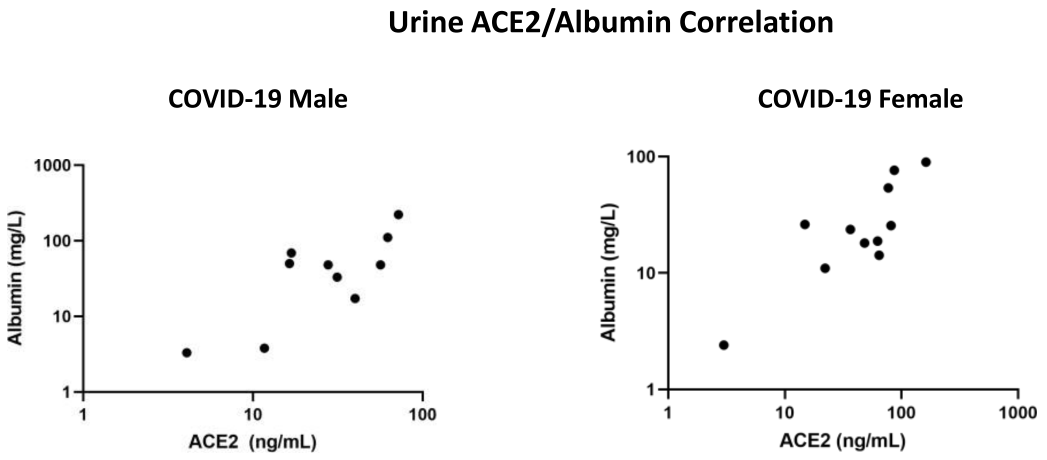

Supplementary figure 7: Correlation between urine ACE2 (ng/mL) and albumin (mg/L) in male and female patients infected with severe SARS-CoV-2. Correlation analysis demonstrated that urine ACE2 and albumin positively and significantly correlate in both male (r^2=0.7873, p=0.069) and female (r^2=0.8540, p=0.0004)**.** The data was log-transformed to yield normaldistribution and plotted on a double logarithmic scale. Covid-19 infected Men n=7, women n=11, non-infected n=6 and KTRs n=12

Supplementary Figure 8

a

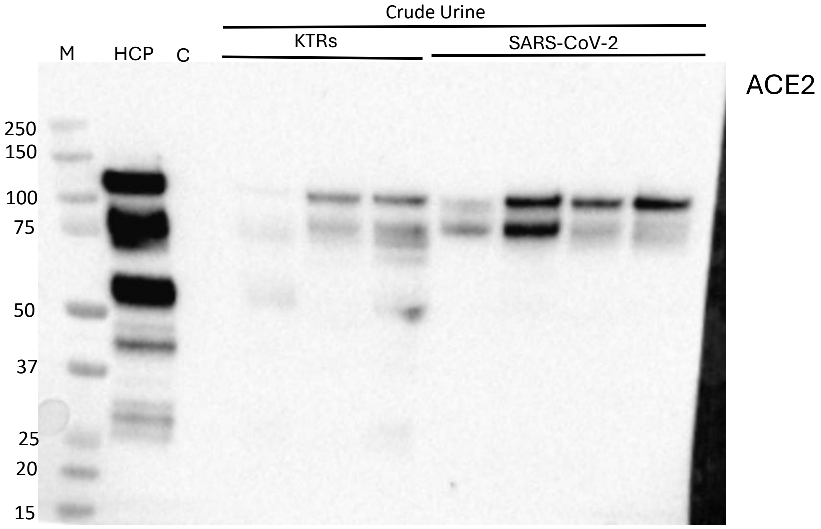

b

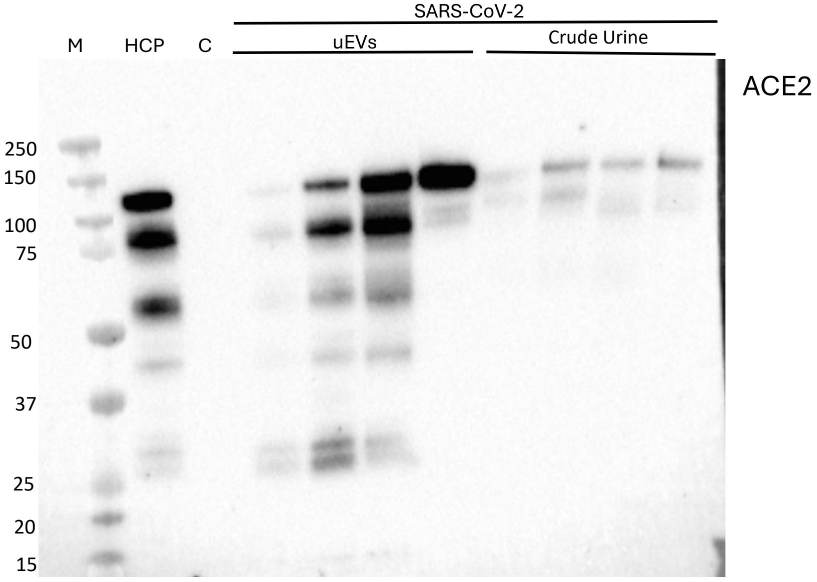

Supplementary figure 8: Non-cropped immunoblots for the detection of ACE2 protein in a**)** crude urine from kidney transplant recipients (KTRs) and COVID-19 patients, and b**)** crude urine vs PEG precipitated EVs from COVID-19 patients. Human cortex pool (HCP*) was used as positive control. M, Marker. *HCP was lysed with a different lysis buffer compared to the HCP used in previous blots.
